# Preferential sugar utilization by bifidobacterial species

**DOI:** 10.20517/mrr.2023.19

**Published:** 2023-08-18

**Authors:** Ryuta Murakami, Keisuke Yoshida, Mikiyasu Sakanaka, Tadasu Urashima, Jin-Zhong Xiao, Takane Katayama, Toshitaka Odamaki

**Affiliations:** ^1^Next Generation Science Institute, R&D Division, Morinaga Milk Industry Co., Ltd., Kanagawa 252-8583, Japan.; ^2^Graduate School of Biostudies, Kyoto University, Kyoto 606-8502, Japan.; ^3^Department of Food and Life Science, Obihiro University of Agriculture and Veterinary Medicine, Obihiro, Hokkaido 080-8555, Japan.

**Keywords:** *Bifidobacterium*, preferential utilization, carbon source, human-residential bifidobacteria, lactose

## Abstract

**Aim:** Bifidobacteria benefit host health and homeostasis by breaking down diet- and host-derived carbohydrates to produce organic acids in the intestine. However, the sugar utilization preference of bifidobacterial species is poorly understood. Thus, this study aimed to investigate the sugar utilization preference (i.e., glucose or lactose) of various bifidobacterial species.

**Methods:** Strains belonging to 40 bifidobacterial species/subspecies were cultured on a modified MRS medium supplemented with glucose and/or lactose, and their preferential sugar utilization was assessed using high-performance thin-layer chromatography. Comparative genomic analysis was conducted with a focus on genes involved in lactose and glucose uptake and genes encoding for carbohydrate-active enzymes.

**Results:** Strains that preferentially utilized glucose or lactose were identified. Almost all the lactose-preferring strains harbored the lactose symporter *lacS* gene. However, the comparative genomic analysis could not explain all their differences in sugar utilization preference. Analysis based on isolate source revealed that all 10 strains isolated from humans preferentially utilized lactose, whereas all four strains isolated from insects preferentially utilized glucose. In addition, bifidobacterial species isolated from hosts whose milk contained higher lactose amounts preferentially utilized lactose. Lactose was also detected in the feces of human infants, suggesting that lactose serves as a carbon source not only for infants but also for gut microbes *in vivo*.

**Conclusion:** The different sugar preference phenotypes of *Bifidobacterium* species may be ascribed to the residential environment affected by the dietary habits of their host. This study is the first to systematically evaluate the sugar uptake preference of various bifidobacterial species.

## INTRODUCTION

Bifidobacteria are anaerobic gram-positive bacteria that inhabit the digestive tracts of humans and other animals^[1-3]^. This genus is highly diverse and comprises more than 110 taxa with strong host specificity in their habitats. This specificity is thought to result from their adaptive and evolutionary fitness in diverse environments, including the intestinal tract of their hosts. For instance, some bifidobacterial species that colonize the human infant gut preferentially degrade human milk oligosaccharides (HMOs)^[4,5]^, whereas those that colonize the adult intestine preferentially utilize complex polysaccharides, such as plant-derived dietary fiber^[6-8]^. Additionally, some studies have reported that bifidobacterial strains isolated from the guts of insects have a metabolic pathway for assimilating trehalose, a sugar present in insects, including honeybees, and in plants and fungi^[9,10]^.

Microbial adaptation to the host diet has been reported in several species, including *Lactiplantibacillus plantarum*, *Bacteroides thetaiotaomicron*, and *Bifidobacterium*^[11,12]^. *Phocaeicola plebeius*, which has an enzyme that degrades seaweed-derived polysaccharides, inhabits the gut of Japanese people who have a long history of consuming seaweed but not the gut of North American people who have no such history^[13]^. Adaptation to the host diet, the primary source of essential nutrients for microbial growth, may be considered a survival strategy for gut microbes to outcompete other bacteria in the intestine where nutrient sources are limited.

In environments with multiple saccharides, bacteria can either co-metabolize different carbon sources or preferentially utilize specific compounds to optimize and fine-tune energy metabolism. This preference is referred to as carbon catabolite repression (CCR), which was first described as a diauxie phenomenon in *Escherichia coli* by Monod *et al.*^[14]^. The bacterium imports glucose first before lactose when both sugars are present in the medium. The CCR mechanism was initially attributed to transcriptional regulation mediated by the cyclic AMP receptor protein^[15]^ but is now linked to glucose uptake *via* the phosphoenolpyruvate:sugar phosphotransferase system, which effectively excludes other saccharides from being imported^[16,17]^. In general, glucose is preferentially utilized by bacteria, including *Bacillus subtilis* and *Staphylococcus aureus*^[18]^. Conversely, some bacteria exhibit a reverse CCR mechanism, preferring alternative carbon sources over glucose^[19]^. For instance, the soil-dwelling bacterium *Pseudomonas* preferentially utilizes organic acids, which are abundant in its habitat, over glucose. *Bifidobacterium longum*, which is frequently found in infant feces, has a unique preference for lactose over glucose^[20,21]^. These studies indicate that sugar preferences vary among bacterial species. However, the sugar utilization preference of bifidobacterial species is poorly understood.

In this study, we aimed to systematically analyze the sugar preferences of bifidobacterial species using glucose and lactose as substrates. Then, we used comparative genomic approaches to elucidate the molecular mechanisms underlying species-dependent carbohydrate preferences. Although we failed to identify the genetic traits responsible for this preference, this systematic study provides novel insights into how different species belonging to the same genus adapt differently to the host environment.

## METHODS

### Bacterial strains and media

Eighty-one bifidobacterial strains were purchased from the Riken BioResource Research Center (JCM, Tsukuba, Japan), American Type Culture Collection (ATCC), and German Collection of Microorganisms and Cell Cultures (DSMZ) and used to inoculate MRS medium (Becton Dickinson and Company). After several passages, 40 strains that showed substantial growth in the medium were selected for this study, considering their taxonomic variety. Furthermore, five commercial strains of *Bifidobacterium longum*, *Bifidobacterium bifidum*, and *Bifidobacterium breve* were included. In total, 45 strains of bifidobacteria were used for sugar consumption analyses. Their scientific names, strain numbers, and isolation sources are listed in Table 1.

**Table 1:** List of strains used in this study

**Number**	**Strain**	**Phylogenomic group**	**Origin**	**Classification**
1	*B. psychraerophilum* JCM 15958	*B. psychraerophilum*	Porcine cecum	Pig
2	*B. asteroides* JCM 8230	*B. asteroides*	Hindgut of honeybee	Bee
3	*B. coryneforme* JCM 5819	*B. asteroides*	Hindgut of honeybee *Apis mellifera caucasica*	Bee
4	*B. callimiconis* JCM 33352	*B. tissieri*	Stool sample of a Goeldi’s marmoset (*Callimico goeldii*)	Marmoset
5	*B. simiarum* JCM 31793	*B. tissieri*	Feces of an adult emperor tamarin (*Saguinus imperator*)	Tamarin
6	*B. tissieri* JCM 30798	*B. tissieri*	Feces of a common marmosets	Marmoset
7	*B. avesanii* JCM 30943	Not categorized 1	Feces of an adult subject of the cotton-top tamarin	Tamarin
8	*B. boum* ATCC 27917	*B. boum*	Rumen of cattle	Others
9	*B. porcinum* JCM 16945	*B. boum*	Piglet feces	Pig
10	*B. thermacidophilum* ssp. *thermacidophilum* JCM 11165	*B. boum*	Wastewater of a bean-curd farm	Others
11	*B. thermophilum* ATCC 25525	*B. boum*	Swine feces	Pig
12	*B. mongoliense* JCM 15461	Not categorized 2	Airag, a traditional fermented mare’s milk product from Mongolia	Others
13	*B. subtile* ATCC 27537	Not categorized 2	Sewage	Sewage
14	*B. aemilianum* JCM 33373	*B. bombi*	Gut samples of carpenter bees (*Xylocopa violacea*)	Bee
15	*B. bohemicum* JCM 18049	*B. bombi*	Digestive tract contents of a bumblebee (*Bombus lucorum*)	Bee
16	*B. minimum* JCM 5821	Not categorized 3	Sewage	Sewage
17	*B. tsurumiense* JCM 13495	Not categorized 3	Dental plaque from hamster fed with a high-carbohydrate diet	Others
18	*B. breve* JCM 1192	*B. longum*	Intestine of infant	Human
19	*B. breve* MCC1274	*B. longum*	Intestine of infant	Human
20	*B. breve* MCC1851	*B. longum*	Intestine of infant	Human
21	*B. felsineum* JCM 31789	*B. longum*	Feces of an adult subject of the cotton-top tamarin (*Saguinus oedipus*)	Tamarin
22	*B. longum* ssp. *infantis* ATCC 15697	*B. longum*	Intestine of infant	Human
23	*B. longum* ssp. *infantis* MCC1872	*B. longum*	Intestine of infant	Human
24	*B. longum* ssp. *longum* JCM 1217	*B. longum*	Intestine of adult	Human
25	*B. longum* ssp. *longum* MCC5360	*B. longum*	Intestine of infant	Human
26	*B. parmae* JCM 32706	*B. longum*	Stool sample of a pygmy marmoset (*Callithrix pygmaea*)	Marmoset
27	*B. reuteri* JCM 17295	*B. longum*	Feces of a common marmoset	Marmoset
28	*B. saguini* JCM 17297	*B. longum*	Feces of a red-handed tamarin	Tamarin
29	*B. stellenboschense* JCM 17298	*B. longum*	Feces of a red-handed tamarin	Tamarin
30	*B. aerophilum* JCM 30941	*B. bifidum*	Feces of an adult subject of the cotton-top tamarin	Tamarin
31	*B. biavatii* JCM 17299	*B. bifidum*	Feces of a red-handed tamarin	Tamarin
32	*B. bifidum* MCC2030	*B. bifidum*	Intestine of infant	Human
33	*B. hapali* JCM 30799	*B. bifidum*	Feces of a common marmosets	Marmoset
34	*B. ramosum* JCM 30944	*B. bifidum*	Feces of an adult subject of the cotton-top tamarin	Tamarin
35	*B. adolescentis* JCM 1275	*B. adolescentis*	Intestine of adult	Human
36	*B. dentium* DSMZ20436	*B. adolescentis*	Dental caries	Human
37	*B. pseudocatenulatum* JCM 1200	*B. adolescentis*	Feces of an infant	Human
38	*B. pullorum* ssp*. gallinarum* JCM 6291	*B. pullorum*	Chicken cecum	Bird
39	*B. pullorum* ssp*. saeculare* JCM 8223	*B. pullorum*	Rabbit feces	Others
40	*B. animalis* ssp. *animalis* ATCC 25527	*B. pseudolongum*	Rat feces	Others
41	*B. animalis* ssp. *lactis* DSMZ10140	*B. pseudolongum*	Yogurt	Others
42	*B. anseris* JCM 32705	*B. pseudolongum*	Stool sample of a domestic goose (*Anser domesticus*)	Bird
43	*B. choerinum* ATCC 27686	*B. pseudolongum*	Swine feces	Pig
44	*B. choloepi* JCM 34854	*B. pseudolongum*	Feces of an adult subject of the two-toed sloth	Others
45	*B. pseudolongum* ssp. *pseudolongum* JCM 1205	*B. pseudolongum*	Swine feces	Pig

### Bacterial culture

Preferential utilization of glucose or lactose was assessed as previously described^[20]^. Modified MRS medium [1% w/v proteose peptone #3, 1% w/v beef extract, 0.5% w/v yeast extract (all from Becton Dickinson and Company Franklin Lakes, NJ, USA), 0.1% v/v Tween 20 (Nacalai Tesque Inc., Tokyo, Japan), 0.5% w/v K_2_HPO_4_ (Kokusan Chemical Co., Tokyo, Japan), and 0.5% w/v sodium acetate, 0.5% w/v di-ammonium hydrogen citrate, 0.02% w/v MgSO_4_·7H_2_O, and 0.005% MnSO_4_·H_2_O (all from Fujifilm Wako Pure Chemical Corporation, Tokyo, Japan), pH 6.5] was used for cultivation. The medium was supplemented with 0.8% (w/v) glucose (MRS-G), 0.8% lactose (MRS-L), or a mixture of 0.4% glucose and 0.4% lactose (MRS-GL) [Supplementary Table 1] prior to inoculation. We employed these sugar concentrations for determining the sugar preference of each strain because we found that these concentrations enable us to visually monitor the sugar consumption by high-performance thin-layer chromatography (HPTLC) analysis (see below).

Bacteria were pre-cultured in MRS medium at 37 °C for 24 h under anaerobic conditions, and then the cultures (400 µL per each) were centrifuged at 2,000 g for 3 min. The resulting pellets were washed twice with a saline solution (Otsuka Pharmaceutical, Tokyo, Japan), and 75 µL of each of the 400 µL suspensions was used to inoculate 2.5 mL of MRS-GL, MRS-G, or MRS-L medium supplemented with 0.05% (w/v) L-cysteine HCl (Kanto Chemical, Tokyo, Japan) in a 5 mL tube (Sarstedt, Nuembrecht, Germany). The culture broth was incubated at 37 °C for 72 h under anaerobic conditions (in N_2_:H_2_:CO_2_, 80:10:10). Aliquots (0.2 mL per each) were taken and the optical densities at 600 nm (OD_600_) were measured using a multi-microplate reader (SH-9000Lab, Hitachi, Tokyo, Japan). The cultures were centrifuged, and the resulting supernatants were used for high-performance thin-layer chromatography (HPTLC).

### HPTLC analysis

Pre-coated silica gel-60 HPTLC aluminum plates (Merck, Darmstadt, Germany) were used to evaluate the sugar preferences of the bifidobacterial strains. The culture supernatants (0.5 µL per each) were loaded on the plate. The spotted plate was developed in a solvent system consisting of 1-butanol (Nacalai Tesque):acetic acid (Kanto Chemical):water (2:1:1 v/v). The plates were stained by spraying with diphenylamine-aniline-phosphoric acid reagent as previously described^[22]^ , and the sugars were visualized by baking the plates for 2 min.

### Comparative genomic analysis

Genomic information of the type strains of the 40 *Bifidobacterium* species was retrieved from the NCBI public database [Supplementary Table 1]. Given its preference for lactose over glucose by repressing the glucose transporter glcP in the presence of lactose in the medium^[20]^, *B. longum* NCC2705 was also included in this *in silico* analysis as the strain has been shown to exhibit a preference for lactose over glucose by repressing the glucose transporter glcP in the presence of lactose in the medium^[20]^. Amino acid sequence comparisons were performed using all-against-all bidirectional BLAST alignment (cut-off: E-value 0.0001, with at least 50% identity across at least 50% of either protein sequence). The sequences were then clustered into protein families by using the Markov clustering algorithm (MCL) implemented in the mclblastline pipeline v12-0678^[23]^. An example of the scripts used for the analysis is available at https://github.com/frbot/Comparative-genomics/blob/master/comparative.sh. The upstream sequence of *glcP* was aligned using the ClustalW alignment tool^[24]^ with default parameters. The carbohydrate-active enzymes were annotated using the dbCAN3 server (https://bcb.unl.edu/dbCAN2/) and the HMMER: dbCAN tool (E-Value < 1e-15, coverage > 0.35). Intergroup differences were analyzed using Welch’s *t*-test, followed by Benjamini-Hochberg FDR calculations on R software (ver. 3.6.0) with the qvalue package (http://www.bioconductor.org/packages/release/bioc/html/qvalue.html).

### Fecal sample collection

Fecal samples of 10 infants at 2, 4, 6, and 8 weeks of age were collected from their diapers by their parents as soon as possible after defecation. The samples were stored at -20 °C at home until all samples from each subject were collected. The samples were then transported to the laboratory at less than -18 °C by logistics companies. Upon receipt, the samples were immediately stored at -80 °C until use. This study was approved by the institutional review board of the Japan Conference of Clinical Research (approval number: BONYU-01) and was conducted in accordance with the latest Declaration of Helsinki in 2013. Healthy mothers of the infants provided written informed consent for their participation in all procedures associated with the study.

### Determination of lactose content in infant fecal samples

Lactose in the fecal samples was fluorescently labeled and quantified using high-performance liquid chromatography (HPLC). Specifically, the lyophilized fecal samples (3-5 mg) were rehydrated with 5% acetic acid (100 μL), fragmented in an ultrasonic bath for 10 min, and then mixed with a solution containing 120 µL of chloroform (Nacalai Tesque Inc.), 240 µL of methanol (Nacalai Tesque Inc.), and 20 µL of 10 mM melibiose (Fujifilm Wako Pure Chemical Corporation). Melibiose was used as the internal standard. After further mixing with 120 µL of chloroform and 120 µL of pure water (Milli-q), the supernatant obtained by centrifugation (21,500 g, 3 min, 4 ºC) was lyophilized after evaporation of methanol. The saccharides were then fluorescently labeled with 2-anthranilic acid and purified by solid extraction using Discovery DPA-6S columns (50 mg) (Supelco, Poole, UK) following a previously described method^[25]^. HPLC was performed using a Thermo U3000 system (Thermo Fisher Scientific, Waltham, MA, USA) equipped with a TSKgel Amide-80 HR column (4.6 mm × 250 mm, φ = 5 μm) (Tosoh, Tokyo, Japan) as previously described^[26]^. The labeled sugars were detected using a Waters 2475 Fluorescence Detector (Waters Corp., Milford, MA, USA). Lactose concentrations were calculated using known concentrations of standard lactose and normalized to the internal standard melibiose.

## RESULTS

### Preferential utilization of glucose or lactose by each bifidobacterial strain

**Figure 1:**
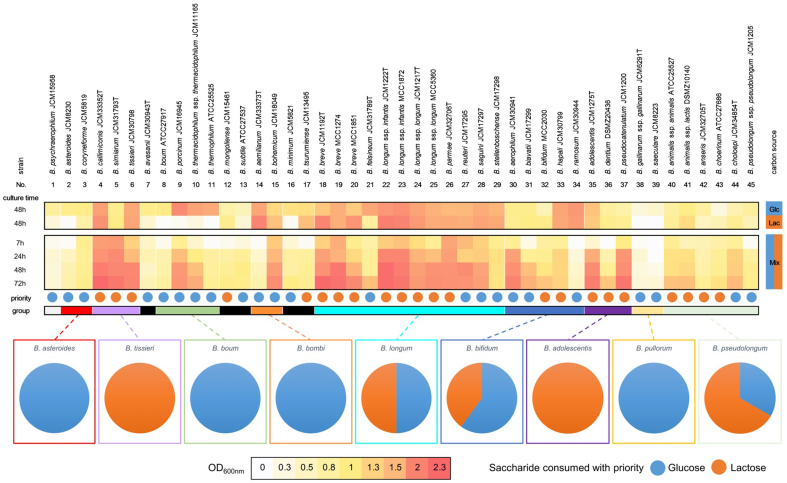
Saccharide utilization preferences and the ability of each *Bifidobacterium* strain to grow on each carbon source. Strains are sorted based on concatenation of 101 core protein sequences of the genus *Bifidobacterium* as previously reported. Numbering of each strain corresponds to Table 1.

**Figure 2:**
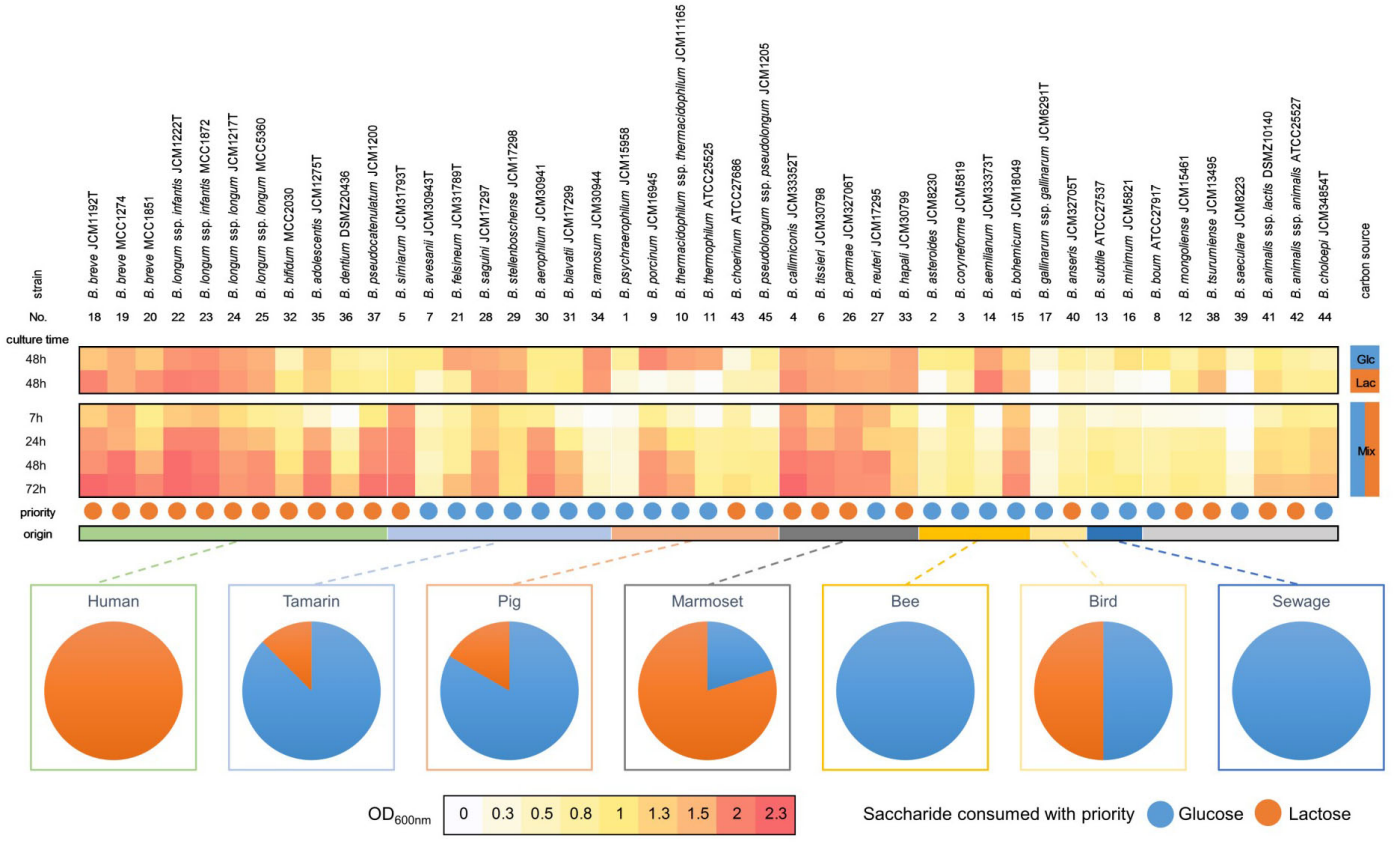
Preference of glucose and lactose utilization and the ability of each *Bifidobacterium* strain to grow on each carbon source. Strains are sorted based on their isolation source.

The ability of each *Bifidobacterium* strain to grow in the MRS-G and MRS-L media was evaluated [Figure 1]. Although most strains were able to utilize both glucose and lactose, the strains of *B. asteroides* (#2, the numbers are indicated in Table 1 and Figures 1 and 2)*, B. boum* (#8), *B. thermophilum* (#11), *B. pullorum* subsp. *gallinarum* (#38), and *B. pullorum* subsp. *saeculare* (#39) showed limited growth on a lactose-containing medium (OD_600_ < 0.1). In general, the strains of *B. boum* group and *B. pollurum* group showed poor lactose utilization. The preferential carbon utilization of each strain was then evaluated by incubating the strains in a medium containing both glucose and lactose and analyzing their sugar consumption behavior through HPTLC [Supplementary Figures 1 and 2]. The results definitively differentiated lactose- and glucose-preferring strains. Based on the phylogenomic tree of the genus *Bifidobacterium* reported by Lugli *et al.*, we found that the *B. asteroides*, *B. boum*, *B. bombi*, and *B. pullorum* groups preferentially utilized glucose, whereas *B. tissieri* and *B. adolescentis* groups preferentially utilized lactose^[27]^. The *B. longum*, *B. bifidum*, and *B. pseudolongum* groups contained glucose- and lactose-preferring strains. Evaluations were also conducted using commercialized strains belonging to three taxa: *B. breve* (#19 and 20), *B. longum* subsp. *longum* (#25), and *B. longum* subsp. *infantis* (#23), with no differences in sugar preference observed within the species or subspecies.

### Distribution of genes involved in lactose and glucose uptake among bifidobacterium species

The distribution of the lactose symporter gene *lacS*^[28]^ was examined to explore the genetic basis of preferential sugar utilization by bifidobacteria. All of the strains that showed a preference for lactose over glucose had a *lacS* homolog with amino acid identities higher than 50%, except for *B. choerinum* ATCC 27686 (#43) [Supplementary Table 2]. The exceptional strain *B. choerinum* ATCC 27686 (#43), whose genomic sequence was complete, showed the lowest growth rate in the medium supplemented with glucose. A *lacS* homolog search also revealed that not all strains with a *lacS* homolog preferentially utilized lactose. For example, *B. reuteri* JCM 17295 (#27) and *B. pseudolongum* ssp*. pseudolongum* JCM 1205 (#45) preferred glucose over lactose. Next, we investigated the homolog distribution of the *glcP*, *ptsG*, and *licT* gene cluster (BL1631, BL1632, and BL1633 in *B. longum* NCC2705), which are reportedly involved in the preferential utilization of lactose in NCC2705^[20]^. Of the 40 isolates, 12 strains had all three homologs, and six had three genes within the same cluster. However, we found no correlation between the presence of these genes and the preferential utilization of lactose [Supplementary Table 2]. DNA sequence alignment was conducted for the regulatory regions of *glcP*, which comprise a potential rho-independent terminator and a ribonucleic anti-terminator (RAT) element, to identify differences that could explain the variability in the preferential utilization of sugars. However, no distinguishing features were detected [Supplementary Figure 3]. Gene-trait matching based on MCL clustering did not reveal any corresponding gene family [Supplementary Table 3]. We also examined the distribution of glycosyl hydrolases using dbCAN; however, no clear relationship was detected between the preferential sugar utilization phenotype and the presence of these enzymes [Supplementary Table 4].

### Link between sugar preference by bifidobacterial strains and their isolation source

The relationship between the origin of the bifidobacterial isolates and their sugar preferences was investigated by sorting them based on their source [Figure 2, Supplementary Figures 1 and 2]. All human-derived bifidobacterial strains belonging to seven species/subspecies demonstrated a preference for lactose, whereas all strains isolated from bees and sewage exhibited a preference for glucose. The other bifidobacterial strains isolated from different sources showed varied preferences for glucose and lactose; however, four of the five strains isolated from marmosets preferred lactose. In addition, *B. animalis* ssp. *lactis* (#41) and *B. mongoliense* (#12) isolated from yogurt and horse milk wine, respectively, preferentially utilized lactose.

### Relationship between lactose content in host milk and lactose-preferring bifidobacterial strains

Considering that *B. animalis* ssp. *lactis* and *B. mongoliense* are commonly found in lactose-rich environments, we hypothesized that the lactose content in the milk of their host animals is associated with their preference for lactose utilization. A literature search was conducted to obtain data on the lactose content in mammalian milk [Table 2]. The lactose concentration in the breast milk of humans and marmosets was relatively higher (≥ 75 g/L) than that in the milk of other animals (< 75 g/L), and most strains isolated from humans and marmosets showed a relatively strong preference for lactose [Figure 2 and Supplementary Figure 4].

**Table 2:** Lactose concentrations in host animals’ milk, as obtained from published studies

**Animal**	**Lactose (g/L)**	**Sample number**	**Ref.**
Human	78 ± 8.8	273	[29]
	79.2 ± 11	412	[30]
Marmoset	75	-	[31]
	78 (reared), 80 (wild)	-	[32]
	80	-	[33]
Tamarin	72	-	[34]
Pig	48	-	[35]
	45	18	[36]
	55	-	[37]
Rat	11 ± 0.23 - 33 ± 1.82	27	[38]
	26	-	[37]
Hamster	49	-	[37]
Cow	36.1 ± 13	10	[39]
Rabbit	19, 14-26	-	[40]

### Lactose contents in the fecal samples of breastfed infants

**Figure 3:**
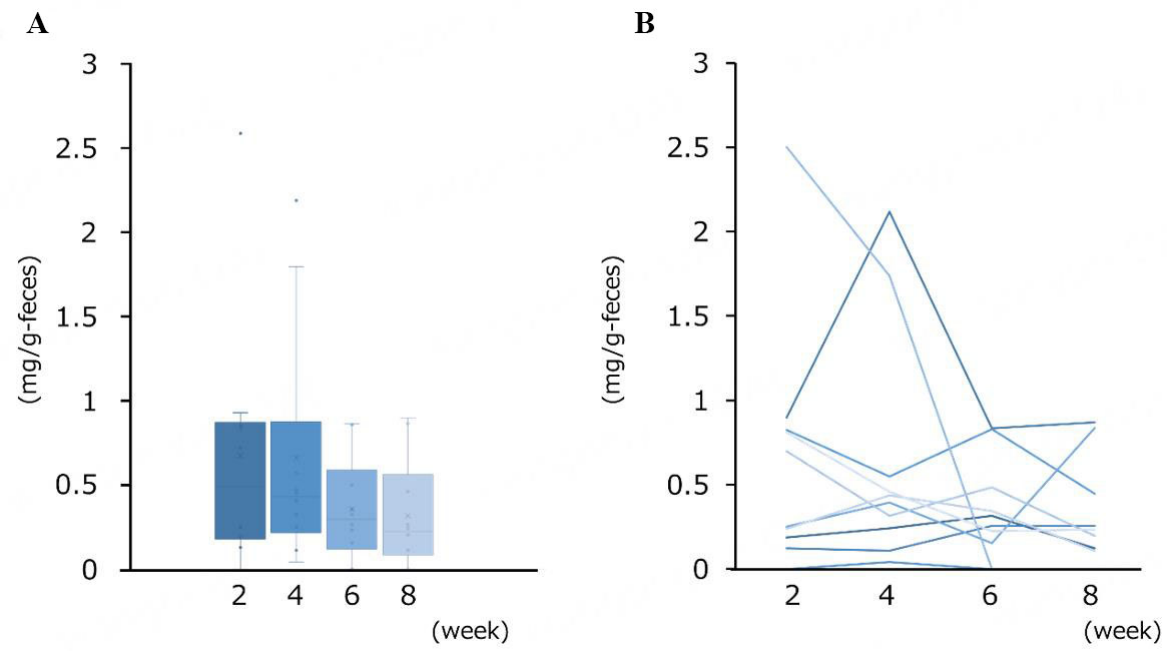
Lactose concentration in fecal samples collected from 10 breastfed infants at 2, 4, 6, and 8 weeks of age. (A) Boxplots of lactose concentrations. Medians with interquartile range are shown. The whiskers represent quartile range; (B) Changes in fecal lactose concentrations in individuals over 6 weeks.

Finally, to infer the lactose availability in the gut microbial ecosystem of human infants, lactose concentrations were determined in fecal samples obtained from 10 breastfed infants at 2, 4, 6, and 8 weeks of age. Lactose was detected in 37 out of 40 samples, with median concentrations of 0.47, 0.42, 0.29, and 0.22 mg/g feces at the respective time points [Figure 3].

Upper heatmap displays the optical density (OD_600nm_) values of *Bifidobacterium* cultures in MRS medium supplemented with 0.8% w/v glucose or lactose. Lower heatmap shows the OD_600nm_ values of *Bifidobacterium* cultures in MRS medium supplemented with a mixture of 0.4% w/v glucose and 0.4% w/v lactose. Colored bars indicate the phylogenetic group to which each strain belongs. Black bars indicate the strains that were not classified into any group. Pie charts represent the ratios of glucose- (blue) and lactose-preferred (orange) strains within the respective taxonomic groups.

This figure is a reorganization of Figure 1 based on the isolation source rather than the phylogenetic group. The light gray bar indicates strains that were not classified into any group. The number of each strain is listed in Table 1.

## DISCUSSION

Bifidobacteria play a crucial role in the human gut, particularly in breastfed infants, where they contribute to healthy development by producing short-chain fatty acids and tryptophan metabolites, such as indole-3-lactic acid^[41,42]^. In this study, we investigated the sugar utilization preference of 40 bifidobacterial species/subspecies that colonize diverse hosts, based on a previous finding that *B. longum* NCC2705 preferentially metabolizes lactose over glucose^[20]^. The results of the *in vitro* assay demonstrated that the sugar preferences of the bifidobacterial species varied. Our results also suggested that sugar preference within the genus *Bifidobacterium* may be linked to the isolation source and phylogenetic status^[27]^. To the best of our knowledge, this study is the first to systematically evaluate the order of sugar uptake in various bifidobacterial species.

All human-residential bifidobacteria (HRB) strains preferentially utilize lactose. *B. longum* subsp. *infantis, B. breve, B. bifidum,* and *B. longum*, which are present in the infant gut, can utilize HMOs^[25]^, and this feature has been linked to their dominance in the infant gut^[5]^. Although lactose is considerably more abundant than HMOs in breast milk, whether lactose would reach the large intestine is unclear, considering that it should be absorbed in the small intestine as an energy source for infants. In the present study, lactose was detected in the feces of all infants, indicating that lactose could also serve as an energy source for gut microbes, particularly for bacteria that preferentially metabolize lactose and have a competitive advantage in the gut of hosts consuming breast milk with high lactose content. The lactose concentration in the infant feces was higher at 2-4 weeks after birth than at 6 weeks. This result suggests that the lactose breakdown and absorption functions in infants are underdeveloped in the first month of life and that lactose in fecal samples is more abundant during this period. Lactose can be released from HMOs by extracellular lacto-N-biosidases^[43,44]^, fucosidases^[45,46]^, and sialidases^[47]^, although the abundance and prevalence of these genes are not generally high in the infant gut microbiome^[4]^. Although the HRB species *B. adolescentis* and *B. dentium* preferentially utilized lactose over glucose, they were not frequently detected in the infant gut. However, *B. adolescentis* is assumed to have evolved over 15 million years in primates^[48]^. Therefore, we speculate that the characteristics acquired during coevolution may have been preserved within the HRB.

Our findings also suggest that bifidobacteria in the gut of breastfed infants utilize lactose, which remains undigested by lactase in the small intestine, in addition to metabolizing HMOs present in breast milk. HMOs in breast milk are present at high concentrations and have various complex chemical structures; thus, their metabolism by infant bifidobacteria may contribute to the formation of a complex, different species/strain-assembled community. Bifidobacteria isolated from the feces of marmosets with high milk carbohydrate concentrations appeared to retain their preferential utilization of lactose. Although the ratio of lactose to milk oligosaccharides in marmoset milk has not been reported, the predominance of lactose in the milk of other New World monkeys, such as capuchin (tufted capuchin), howler (mantled howler), and squirrel monkey (Bolivian squirrel monkey)^[49]^, suggests that marmoset milk contains a high concentration of lactose and a low ratio of milk oligosaccharides. Therefore, bifidobacteria in the intestinal tract of marmosets are expected to grow primarily on lactose rather than on milk oligosaccharides. Bifidobacteria residing in lactating primates possibly first evolved to preferentially assimilate lactose, which is undigested in the small intestine and reaches the large intestine, and then further adapted to the environment in the human gastrointestinal tract by acquiring the ability to metabolize the various chemical structures of HMOs. However, further research is warranted to understand how most bifidobacteria in marmosets differ from those in tamarins, which preferentially utilize glucose rather than lactose.

Four bifidobacterial species isolated from insects preferentially utilized glucose. *B. asteroides*, which effectively metabolizes both glucose and fructose^[50]^, was unable to utilize lactose. *B. animalis* ssp. *lactis* and *B. mongoliense* preferentially utilized lactose and were isolated from fermented cow’s milk and mare’s milk liquor, respectively, both of which contain high levels of lactose in dairy products. These results imply that gene acquisition and loss and/or the CCR system in bifidobacteria are adaptations to their natural habitats.

Searches for genes and gene clusters were conducted to elucidate the mechanisms underlying preferential lactose utilization. Almost all the strains preferentially utilizing lactose harbored a *lacS* homolog [Supplementary Table 2]. However, not all strains with a *lacS* homolog preferentially utilized lactose, and several strains that grew in the lactose-containing medium did not harbor a *lacS* homolog, such as *B. coryneforme* JCM 5819 (#3) and *B. aemilianum* JCM 33373 (#14). Studies have also focused on glycosyl hydrolases, homologs of the *glcP*, *ptsG*, and *licT* gene cluster, and the regulatory regions of *glcP*, which are reportedly involved in the preferential utilization of lactose in *B. longum* NCC2705. However, the results did not reveal any significant genetic traits that could differentiate sugar preference phenotypes. These results suggest that the genetic basis underlying the preference of bifidobacteria for utilizing sugars is complex and possibly involves additional genetic factors that were not examined in this study or have not yet been identified. Some bifidobacterial strains may possess unidentified lactose or glucose transporter(s). For example, *B. choerinum* ATCC 27686 (#43), which showed a preference for lactose over glucose but did not harbor the *lacS* homolog, possesses genes encoding one GH1, two GH2, and two GH42 enzymes that potentially have b-galactosidase activity [Supplementary Table 4]. However, none of the homologs possessed signal sequences at their N-termini. Therefore, this bacterium may possess an unidentified lactose transporter. RNA sequencing analysis and subsequent gene knockout studies using newly developed methods^[6,51]^ are necessary to comprehensively understand the general or species-specific CCR system in the genus *Bifidobacterium*.

This study has some limitations. For instance, the results cannot be generalized to all bifidobacterial species because the evaluation was performed using only strains that grew well in MRS. In addition, in most cases, only type strains were selected for evaluation. Therefore, our results should be interpreted carefully, i.e., whether they are specific to the species or to a certain strain of the species. Furthermore, although trends in sugar preference were observed in accordance with the source of isolation, some results did not align with our hypotheses. For instance, *B. anseris* JCM 32705 (#40) originates from birds and preferentially utilizes lactose. However, birds are not known to consume lactose. The gut microbiota composition is influenced by microbes in the environment^[52]^; therefore, the isolation source does not necessarily reflect the original habitat of the strains. Thus, careful interpretation is necessary to understand how environmental factors affect sugar preferences and adaptation strategies for specific bifidobacterial species. Another limitation of this study is that it did not explore the sugar utilization preference of gut bacteria other than *Bifidobacterium*. Given this competitive environment, the extent to which undigested lactose contributes to bifidobacterial growth in the infant gut remains unclear. From this perspective, further research examining how microbial sugar utilization preferences are regulated and how these preferences contribute to gut microbiota dynamics is warranted.

In summary, the evaluation of the preferential utilization of glucose and/or lactose in *Bifidobacterium* species revealed that most bifidobacterial strains derived from hosts with high lactose content in their milk preferentially utilize lactose. These findings imply that lactose may partly contribute to shaping the early stages of gut microbiota formation and HRB community formation in the infant gut, along with their capacity to metabolize HMOs.

## DECLARATIONS

### Copyright

© The Author(s) 2023.
