# Supplementary material for: Preferential sugar utilization by bifidobacterial species
Source: Microbiome Res Rep. 2023 Aug 18;2(4):31. doi: 10.20517/mrr.2023.19 (PMC10688810; doi:10.20517/mrr.2023.19)
Supplement: Supplementary file 1 [file mrr-2-4-31-SupplementaryMaterials.zip › mrr-2-4-31-SupplementaryMaterials.pdf]

DOI: 10.20517/mrr.xxxx.xx

Microbiome Research Reports

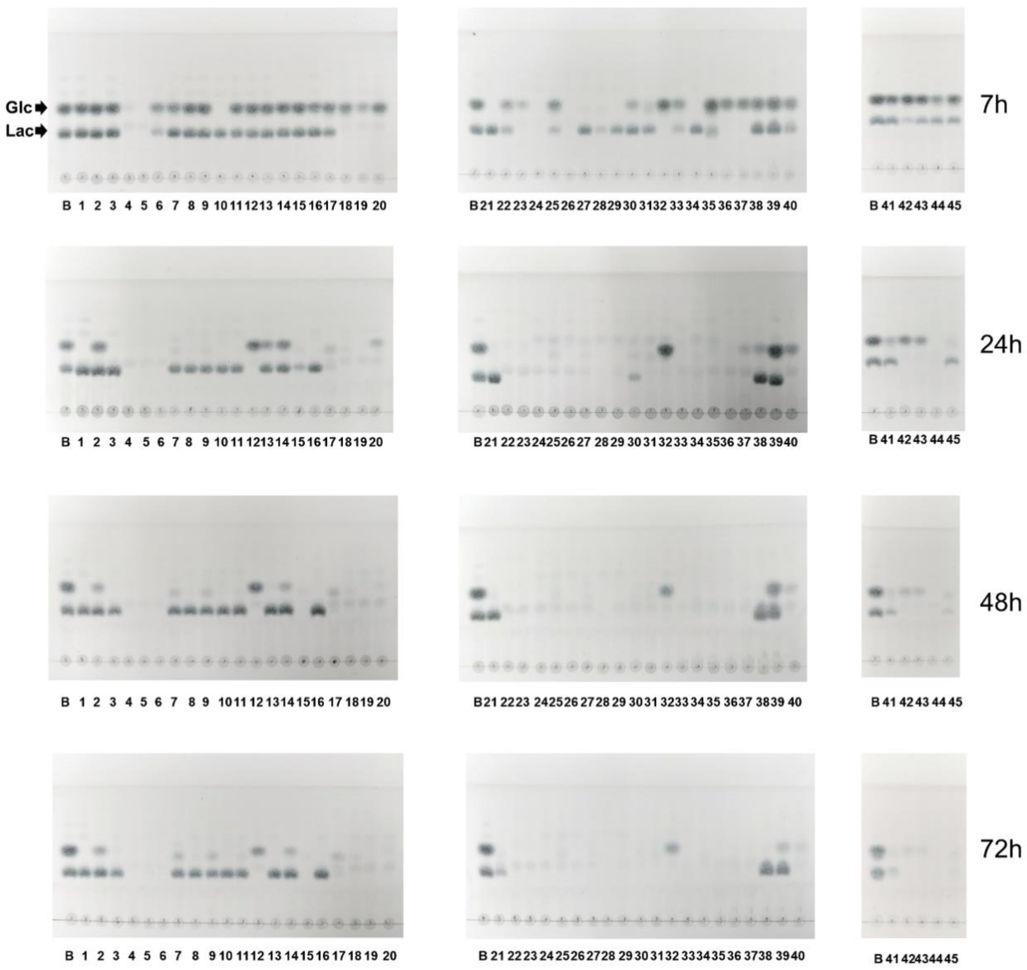

© The Author(s) 2021. Open Access This article is licensed under a Creative Commons Attribution 4.0 International License (https://creativecommons.org/licenses/by/4.0/), which permits unrestricted use, sharing, adaptation, distribution and reproduction in any medium or

format, for any purpose, even commercially, as long as you give appropriate credit to the original author(s) and the source, provide a link to the Creative Commons license, and indicate if changes were made.

OAE Publishing Inc.

www.mrrjournal.net

Supplementary Figure 1. Preferred consumption of glucose and/or lactose by *Bifidobacterium.* Utilization of carbon source by *Bifidobacterium* (No. 1 to 45) was evaluated at different time points using HPTLC. Lane B represents mixed glucose and lactose standard solution.

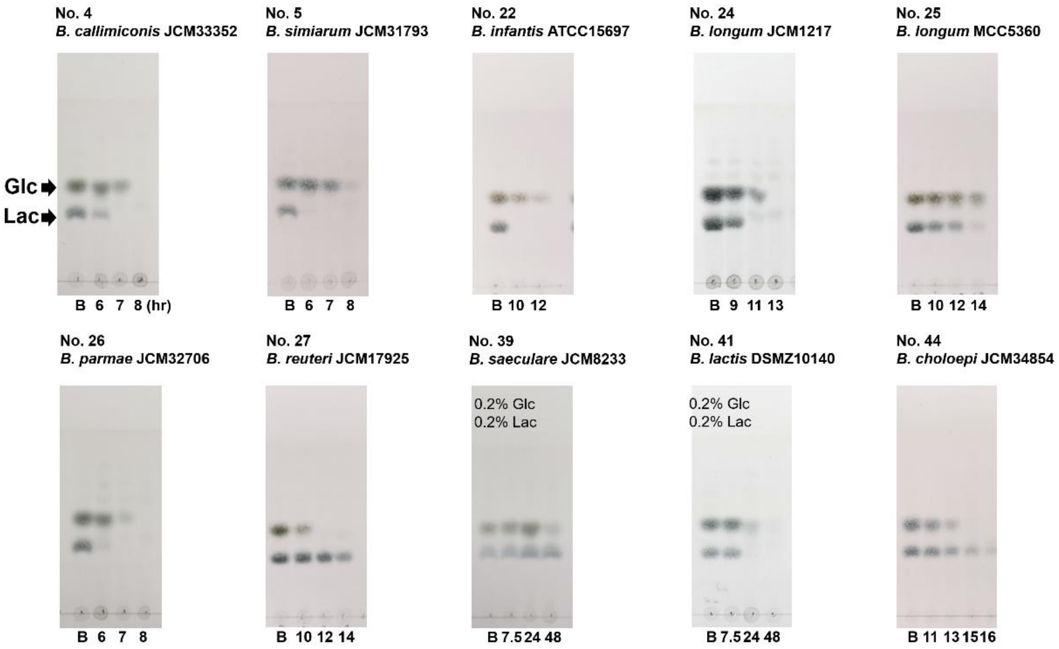

Supplementary Figure 2. Preferential utilization of glucose and lactose by *Bifidobacterium* cultured individually according to their growth rate. Utilization of carbon source by *Bifidobacterium* was evaluated at different time points using HPTLC. Strain Nos. 39 and 41 were cultured with half the concentration of glucose and lactose. Lane B represents mixed glucose and lactose standard solution.

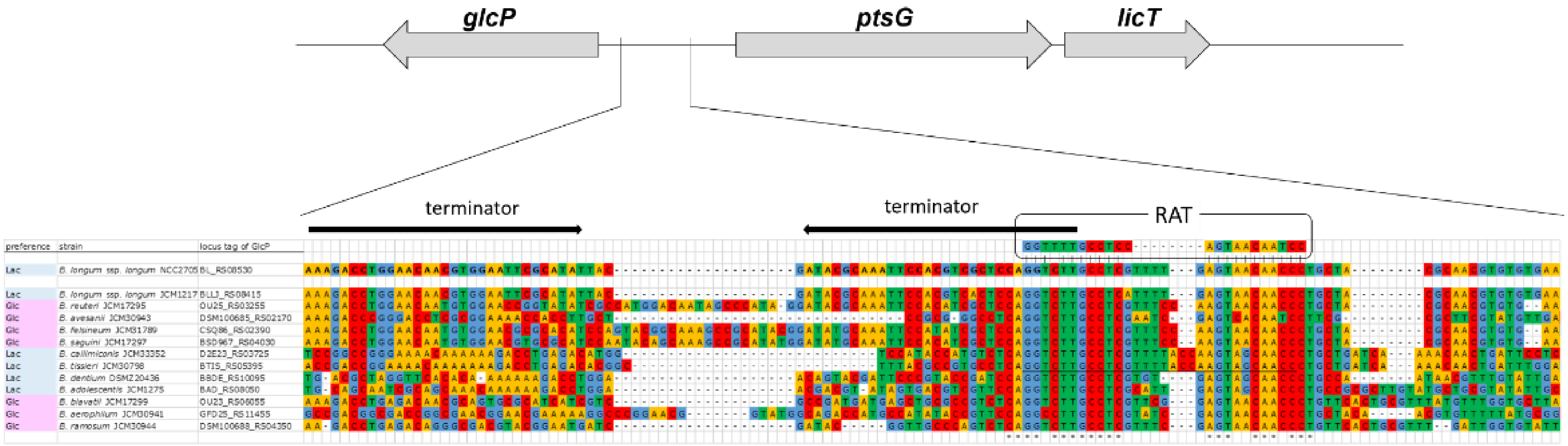

Supplementary Figure 3. Sequence alignment of regulatory region of the *glcP* gene, which comprises a potential rho-independent terminator and a ribonucleic antiterminator (RAT) element, in 13 bifidobacterial strains harboring *glcP*.

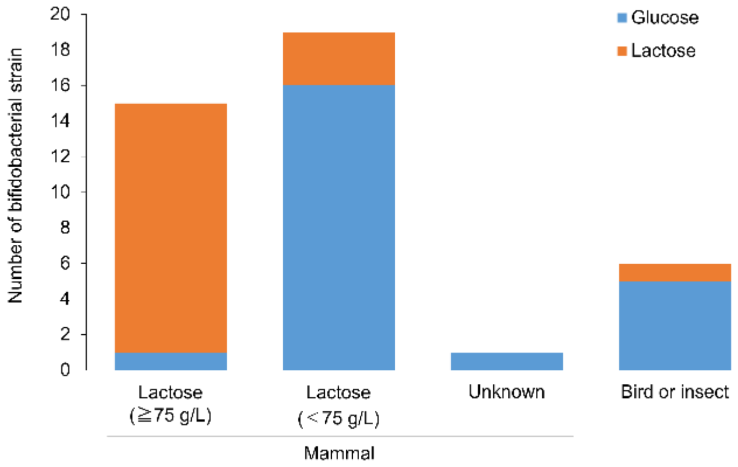

Supplementary Figure 4. Number of bifidobacterial strains preferentially utilizing either glucose or lactose based on lactose content in the milk of their host. High and low lactose indicates milk containing lactose at ≥ 75 g/L or lower than 75 g/L, respectively.
